# Dietary Protein and Amino Acid Supplementation in Inflammatory Bowel Disease Course: What Impact on the Colonic Mucosa?

**DOI:** 10.3390/nu9030310

**Published:** 2017-03-21

**Authors:** Sandra Vidal-Lletjós, Martin Beaumont, Daniel Tomé, Robert Benamouzig, François Blachier, Annaïg Lan

**Affiliations:** 1 UMR 914 INRA/AgroParisTech/Université Paris Saclay, Nutrition Physiology and Ingestive Behavior, 16 rue Claude Bernard, F-75005 Paris, France; sandra.vidal@agroparistech.fr (S.V.-L.); martin.beaumont@agroparistech.fr (M.B.); daniel.tome@agroparistech.fr (D.T.); robert.benamouzig@aphp.fr (R.B.); 2 Department of Gastroenterology, Avicenne Hospital, Assistance Publique-Hôpitaux de Paris, F-93000 Bobigny, France

**Keywords:** inflammatory bowel disease, dietary proteins, amino acid supplementation

## Abstract

Inflammatory bowel diseases (IBD), after disease onset, typically progress in two cyclically repeated phases, namely inflammatory flare and remission, with possible nutritional status impairment. Some evidence, either from epidemiological, clinical, and experimental studies indicate that the quantity and the quality of dietary protein consumption and amino acid supplementation may differently influence the IBD course according to the disease phases. For instance, although the dietary protein needs for mucosal healing after an inflammatory episode remain undetermined, there is evidence that amino acids derived from dietary proteins display beneficial effects on this process, serving as building blocks for macromolecule synthesis in the wounded mucosal area, energy substrates, and/or precursors of bioactive metabolites. However, an excessive amount of dietary proteins may result in an increased intestinal production of potentially deleterious bacterial metabolites. This could possibly affect epithelial repair as several of these bacterial metabolites are known to inhibit colonic epithelial cell respiration, cell proliferation, and/or to affect barrier function. In this review, we present the available evidence about the impact of the amount of dietary proteins and supplementary amino acids on IBD onset and progression, with a focus on the effects reported in the colon.

## 1. Introduction

After disease onset, the alternation of inflammation and remission episodes characterizes the progression of inflammatory bowel diseases (IBD). The two main subtypes of IBD, Crohn’s disease (CD), and ulcerative colitis (UC), which both remain of unclear etiology, are characterized by alterations of the intestinal mucosa homeostasis resulting from inappropriate and exacerbated immune response to intestinal luminal antigens. These chronic unremitting inflammatory diseases often require long-term treatment based on a combination of drugs such as corticosteroids or immunosuppressive compounds in order to control the disease by reducing the inflammatory process. However, such a pharmacological approach has not proved yet to efficiently induce and maintain mucosal healing (MH), which is defined as the complete absence of blood, friability, erosion, and ulcerative lesions in all segments of the gut [1,2]. Regarding this latter aspect, treatment strategies have recently included MH as a clinical endpoint in addition to symptomatic remission [3]. Although IBD patients often ask clinicians for the best dietary practices to avoid relapse and to allow healing, the optimal dietary composition in terms of macronutrients, vitamins, and minerals, as well as the impact of these compounds on the intestinal mucosa are still largely unknown. It is worth noting that the nutritional impact of diet may differ between CD and UC patients [4], the pattern and severity of malnutrition being dependent on the duration, the activity, and the anatomical extent of the disease [5,6]. In fact, malnutrition is more frequently observed in CD patients because the small bowel may be affected, thus causing nutrient malabsorption and possible nutritional deficiencies [7], in contrast to UC, which affects exclusively the colon and rectum. In UC patients, nutritional deficiencies may be likely linked to a reduced oral intake and increased energy requirement due to inflammation [8]. However, the effects of primary nutritional therapy in both CD and UC patients remain largely unknown and need further research. 

**Figure 1:**
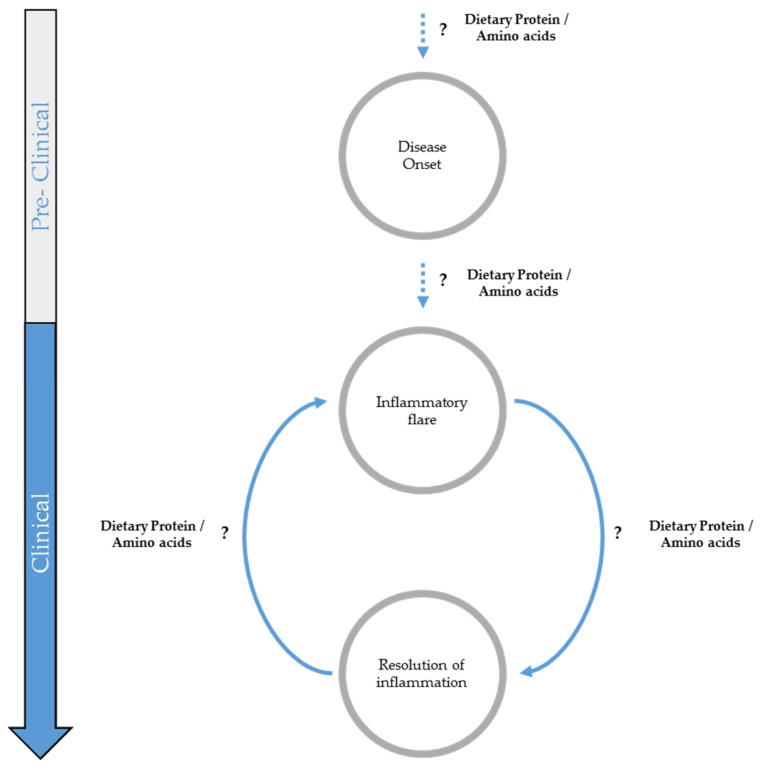
Possible implication of dietary proteins and/or amino acids in inflammatory bowel diseases (IBD) progression.

Nutritional therapy is commonly classified notably by the nitrogen source derived from the amino acid or protein component of the formula. The analysis of the literature, which is based mainly on the results obtained in experimental models but on a limited amount of human studies, reveals that the quality and quantity of dietary proteins are parameters which may have a different functional output depending on the stage of the disease, possibly influencing its course. This lack of robust results obtained from clinical studies resulted in the absence of clear recommendations by the scientific and clinical instances for patients with a recent diagnosis of IBD until recently [9], leading to different nutritional management depending on the clinician involved [10]. Although no formulated food has shown complete efficacy as the sole primary therapy of IBD, current preclinical research focuses on comparing the relative efficacy of different sources and contents of nitrogen on intestinal mucosa. This review therefore aims to provide an update on the reported effects of different types of dietary protein and amino acid (AA) supplementations during the different phases of IBD before and after disease onset (Figure 1) and to illustrate how the modulation of nitrogenous dietary supply may lead to beneficial or detrimental effects for the gut with some insights on possible associated mechanisms. In the present review, we have decided to focus on the effects of dietary protein and amino acid supplementations on the colonic mucosa, taking into account studies on the effects of amino acid-derived bacterial metabolites performed in both normal (healthy) and inflammatory situations.

## 2. Potential Influence of Dietary Proteins on Inflammatory Bowel Disease Onset

Among numerous environmental factors involved in IBD etiology, diet has been suggested to represent an important one in the IBD pathogenesis, notably by altering gut microbiota composition and activity and by participating in mucosal immune system activation. It thus appears that specific dietary patterns may likely be associated with a risk of IBD in adults [11,12]. Indeed, an epidemiologic analysis of CD performed by Shoda et al. showed a correlation between the incidence of IBD and an increased intake of animal proteins over a period of 20 years [13]. A prospective cohort study carried out on women also reported a positive association between the level of protein intake and the risk of IBD [14]. Interestingly in these two studies, this association was found only for animal proteins and not plant proteins [13,14]. Specifically, among the different sources of animal proteins, a high consumption of meat or fish but not eggs or dairy products has been associated with IBD risk [14]. These results suggest that, in addition to quantity, the source of dietary proteins may modulate the risk of developing an IBD. However, Spooren et al. recently performed a systematic review of the epidemiological studies examining the potential link between protein intake and IBD risk, and concluded that more studies (8 out of 10) found no significant association between these two parameters [15]. Due to the complexity of the diet and then to the difficulty of collecting robust dietary data, it is possible that the effects of protein intake on IBD risk observed in only two epidemiological studies [14,16] have been masked by confounding factors in the other studies. However, despite the small number of studies on the high-protein (HP) diet effect in an IBD context, HP intake might influence IBD onset considering its action on intestinal homeostasis, particularly on colonic microbiota and mucosa.

### 2.1. Effect of High-Protein Diets on Microbiota Composition and Activity

Although the digestion of dietary and endogenous proteins is an efficient process with the digestibility of dietary proteins being often higher than 90% [17], parts of luminal proteins may escape digestion in the small intestine and be transferred into the large intestine [18,19]. In fact, the amount of luminal proteins increases according to the quantity of dietary proteins consumed, representing in humans between 6 and 18 g reaching the colon [18]. These nitrogenous substrates are then available for the microbiota activity in the large intestine lumen. Due to differences in AA composition and protein digestibility (animal proteins being generally more digestible than plant proteins [20,21]), protein sources also influence the quantity and nature of AA delivered in the colon [17]. Several animal studies showed that a modification of the protein intake alters the composition of the gut microbiota despite important variations, according to the experimental design. Interestingly, two studies performed with rats receiving an HP diet reported a significant decrease in the abundance of *Faecalibacterium prausnitzii* in the large intestine [22,23]. This commensal bacterium has demonstrated anti-inflammatory properties in vivo using a mouse colitis model. Its abundance has been shown to be reduced in IBD patients [24], but this reduction was not observed in a clinical trial with a HP/low-fiber diet [25]. In this latter study, the dietary intervention reduced instead the abundance of *Roseburia/E. rectale*, which are considered to be health-promoting bacteria, presumably due to their ability to produce butyrate, which has been largely described as a beneficial compound for the colonic mucosa [26]. However, such effects have not been replicated in another human study in which the HP intake was not associated with a decrease in fiber intake [27], suggesting that this latter parameter might be the main driver of changes in microbiota composition. Recent data also suggest that protein sources might regulate the composition of the microbiota [28], but their relevance to gut health remains to be determined. Overall, an increase in protein intake might impact the microbiota composition and induce some typical features of IBD-associated dysbiosis, but more studies, notably with volunteers, are obviously needed to determine the exact contribution of this dietary parameter among others. 

A wide variety of bacterial metabolites can be produced from peptides and AA reaching the colon (for review, see [29]), some amino acid precursors and their main products being presented in Table 1. In humans, HP diets induce a global increase in AA degradation by the microbiota probably through an increase in protein supply and protease activity in the lumen of the large intestine [25,27,30]. Incidentally, a marked increase in protease activity, probably from eukaryotic and prokaryotic origins has been observed in the small and large intestine contents of rats receiving an HP diet [31]. Interestingly, emerging evidences indicate that bacterial proteases in the intestinal lumen might participate to the pathogenesis of IBD [32].

### 2.2. Effect of High-Protein Diet on Colonic Mucosa

Several observations have been made in animal models concerning the effects of HP diet consumption for colonic mucosa health. Although the specific molecular mechanisms associated with such effects are not fully deciphered, there are strong evidences from in vivo and in vitro studies that some bacterial metabolites derived from AA are active towards intestinal epithelial cells (IEC) [33].

**Figure 2:**
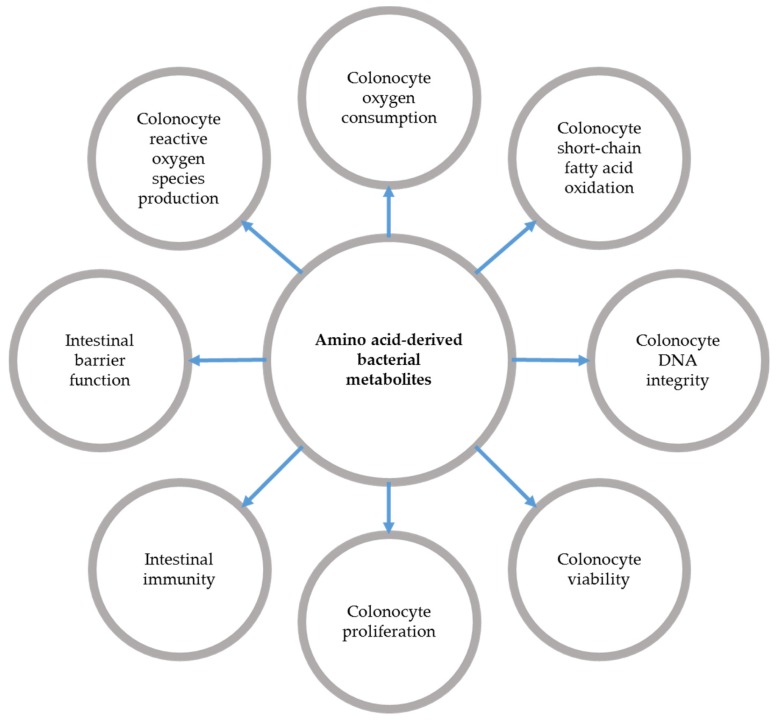
Schematic view of the effects of amino acid-derived bacterial metabolites on colonic epithelial cell physiology and metabolism, and on mucosal homeostasis.

Indeed, among the diversity of bacterial metabolites produced from increased AA degradation, some of them, when present in excess, appear beneficial (butyrate, indole) or detrimental (ammonium, hydrogen sulfide, *p*-cresol) for the colonic mucosa, notably in terms of IEC renewal, colonocyte membrane characteristics, and intestinal barrier function [33] (Figure 2). Such detrimental effects might then participate in an IBD course (see part 3.2). In rats, an HP diet provoked an increase in proton leaks in the mitochondria of colonocytes, then indicating a decrease in the energy metabolism efficiency [31]. Yet, these cells are characterized by high energy consumption because of their constant high anabolic metabolism and ATP consumption. Therefore an impairment of their energy metabolism might be deleterious [34] by altering the physical barrier function. However, the results are rather heterogeneous, depending on the experimental model used. For instance in the pig model, HP diet consumption did not affect the colonic barrier function, despite an alteration in tight junction protein expression [35]. In contrast, it reduced the colonocyte brush-border height in rats [31], indicating that an HP diet may impact the colonic epithelium without affecting barrier function, as we recently reported [36]. Another study also performed on pigs showed that HP diet consumption induced the expression of a proliferation marker in IEC, while having no effect on other apoptosis related parameters [37]. 

Concerning the protective layer of mucus, which covers the intestinal epithelium but which is defective in IBD [38,39], several studies reported that an HP diet increases mucin gene expression in the small and large intestine epithelia of rats and pigs [37,40], probably as a result of an adaptive process towards the modified luminal environment [36]. Interestingly, a study in rats found different effects of an HP intake on the mucus layer according to the protein source [41]. In addition, conflicting results have been obtained on the effects of an HP diet on intestinal immunity. In rats, the HP diet decreased myeloperoxidase activity, toll-like receptor 4, and cytokine gene expression in the ileum, while it virtually did not affect these parameters in the colon [40]. In pigs fed an HP diet, both pro- and anti-inflammatory gene expressions were found to be increased in the colonic mucosa [37]. To summarize, there is no evidence that the consumption of an HP diet induces inflammation or impairs barrier function, at least in animal models. 

Collectively, the data reviewed above indicate that a modification of protein intake is not sufficient to induce an overt perturbation of the intestinal homeostasis that could contribute alone to IBD pathogenesis. This conclusion fits the multifactorial etiology of IBD and the results of most of the epidemiological data, reporting no significant association between protein intake and IBD incidence [15]. However, it is important to consider the short duration of the studies cited above (generally only a few weeks) precludes to conclude on the long-term effects of an HP consumption on gut health.

## 3. Potential Role of Dietary Proteins in Inflammatory Flare

Very few studies reported an association between dietary protein intake and IBD activity. Only one prospective study among 191 patients with UC in remission observed that a high meat/HP/high-sulfur intake was associated with an increased risk of relapse [42]. Furthermore, few animal studies addressed whether the quantity and source of dietary proteins could influence the IBD course. In a rat model of colitis, whey protein improved the clinical symptoms when compared to casein [43], while a diet with red meat as a protein source worsened the disease activity index when compared to a casein based diet in a mouse model of colitis [44]. Nevertheless, it is not possible to exclude that these effects are related to meat components other than protein, like heme, for instance. In another mouse model of colitis, an HP intake (whole milk protein) decreased the survival rate and increased weight loss and the inflammatory score, suggesting an exacerbation of the inflammation during the post-induction colitis phase [45]. 

Repeated intestinal epithelial damage with disruption of the intestinal barrier function is a key feature of IBD [46]. Also, an alteration of the microbiota composition has been repeatedly reported [47] with a notable decrease in the butyrate-producing species in UC patients [48]. Butyrate is a beneficial metabolite mostly produced by the microbiota from indigestible carbohydrates for which transport is deficient in IBD [49]. Moreover, an HP diet decreased the mucosal expression of the butyrate transporter monocarboxylate transporter 1 (MCT1) in pigs [50], but this effect was not observed in rats [22] in different experimental conditions. Then, the impacts of the HP diet described above on the microbiota and colonic epithelium might be amplified in individuals with a compromised barrier function, explaining this increased risk of relapse associated with an HP intake in IBD patients [42]. 

### 3.1. Amino Acid-Derived Bacterial Metabolite Production during Inflammatory Bowel Diseases

Several mechanisms linking microbial dysbiosis to IBD have been proposed [51], but one of the most convincing mechanisms is related to an impairment of the metabolic activity of the gut bacteria that could potentially promote AA degradation and therefore increase the concentration of AA-derived metabolites. Untargeted metabolomics revealed that there is an IBD-associated metabolome in feces [52], and some of this metabolic signature is related to bacterial metabolites derived from AA. Interestingly, while several studies found an increase in AA fecal concentrations in IBD patients [53,54], other studies showed a complex remodeling of AA-catabolism by the microbiota, with some AA-derived bacterial compounds being increased while others decreased. This emphasizes the difficulty in causally linking the modified composition of the bacterial metabolites with the clinical sign of mucosal inflammation. In UC patients, the fecal level of 2-methylbutyrate, a bacterial metabolite produced from isoleucine degradation, was lowered when compared to healthy subjects, and an increase in cadaverine concentration, a bacterial product from lysine degradation, was also measured [55]. Another study found decreased levels of the AA decarboxylation-derived products methylamine and trimethylamine in the feces of IBD patients [53]. Moreover, the host-microbiota ‘co-metabolite’ *p*-cresol sulfate, which is produced from the L-tyrosine derived bacterial metabolite *p*-cresol, was decreased in the urine of CD patients [56]. In accordance with this latter result, another study found decreased *p*-cresol levels in the feces of IBD patients [52]. In this study, the authors found a decreased level of the indolic compounds, which are produced from tryptophan by intestinal bacteria, in accordance with previous results [57]. Targeted measurement of hydrogen sulfide (H_2_S), a bacterial product of cysteine degradation, indicated an increased concentration of this metabolite [58,59], but other studies failed to confirm this finding (reviewed in [60]). Incidentally, the measurement of H_2_S in the colonic luminal content or in feces is a good example of the technical difficulty in measuring accurately the quantity of this gas, especially if the biological samples are not treated immediately after recovery and if the measurement method is not fully specific [61]. 

Interestingly, some AA-derived bacterial metabolites can be used to discriminate IBD subtypes. For instance, a higher concentration of indolic compounds in feces is more related to CD patients than UC [62]. Finally, other metabolites are associated with disease activity. For instance in CD, fecal *p*-cresol concentration is increased during remission [63]. In conclusion, there is strong evidence showing that there is a remodeling of microbial AA catabolism during IBD. However, since some of the AA-derived metabolites are increased while others are decreased, it is difficult to draw general conclusions on the relevance for IBD of AA-degradation by the microbiota. Nevertheless, as described below, data from preclinical studies have shown that bacterial metabolites may have a beneficial or deleterious impact on the intestinal epithelium.

### 3.2. Effects of Bacterial Metabolites Derived from Amino Acids on Intestinal Epithelial Cells

Several AA-derived bacterial metabolites are metabolic troublemakers in IEC, namely H_2_S, *p*-cresol, and ammonia [64,65,66]. The inhibition of mitochondrial respiration induced by *p*-cresol is associated with an increase in superoxide anion production in IEC, probably implicated in the genotoxicity of this compound [64]. Conflicting results were obtained concerning the effects of H_2_S and ammonia on cell proliferation and viability, according to the experimental settings [67,68,69,70]. Polyamines from bacterial and endogenous origins are also important regulators of cell proliferation, some of them inhibiting proliferation while others promote it [71]. Collectively, these results suggest that AA-derived metabolites have an impact on IEC metabolism and potentially on IEC renewal. However, more studies are needed to confirm these effects that might be relevant to the impairment of mucosal integrity observed in IBD. Recently, H_2_S has been proposed to destabilize the mucus layer through disulfide bond reduction [72,73], therefore possibly facilitating the contact of luminal compounds with the colonic mucosa. The AA-derived microbial metabolites phenol and ammonia have been found to increase epithelial permeability [74], while indole and branched-chain fatty acids have shown protective effects on this intestinal parameter [75,76,77]. Among AA-derived bacterial metabolites, ammonia and H_2_S have been shown to induce the expression of pro-inflammatory genes in colonic IEC [50,65] while indole and related compounds exert immuno-regulatory effects [75,76,78,79]. Recently, the bacterial AA-degradation products histamine and spermine have been shown to regulate inflammation in the intestinal epithelium [80]. In addition, some defects in the detoxication system of several detrimental bacterial metabolites derived from AA have been shown in IBD. Indeed, the gene expression and the activity of the H_2_S detoxification-enzyme thiosulfur transferase (TST) are downregulated in the mucosa of IBD patients, and this downregulation is correlated with inflammation [81], notably in CD patients [82]. Similarly, in UC, there is an impaired mucosal sulphation of phenolic compounds produced by the gut microbiota from tyrosine [83]. 

In conclusion, AA-derived bacterial metabolites impact key processes in the IEC metabolism and physiology and presumably in mucosal homeostasis (Figure 2), some of them being detrimental and some others being beneficial. Since an HP intake globally increases the luminal amount of these metabolites, it is difficult to predict the consequences for the colonic mucosa, since the combined effects of these compounds are little known. Indeed, even if those effects may be reproduced by a fecal water test, which evaluates the global effect of water-soluble fecal compounds on IEC, the results remain unconclusive. Importantly, several studies suggest that the decreased capacity of the colonic mucosa to metabolize deleterious AA-derived metabolites during IBD might result in an amplification of their detrimental effects. One promising future strategy would be to rationally select the protein source, taking into account their AA profile and their digestibility in order to specifically promote the production of beneficial AA-derived metabolites. Conversely, the consumption of dietary unabsorbed or partially absorbed compounds able to bind deleterious bacterial metabolites in the colonic luminal content represents a promising strategy for diminishing their free luminal concentration [84,85] and thus their negative impact on the colonic mucosa.

## 4. Potential Role of Dietary Protein Intake and Amino Acid Supplementation in Remission

During an inflammatory flare, IBD patients are at high risk of a nutrient depletion [86,87,88], particularly children that may experience such depletion up to the remission phase [9,88,89]. New guidelines from the European Society for Clinical Nutrition and Metabolism (ESPEN) recommend to increase the protein requirement in active IBD to 1.2–1.5 g/kg body weight/day in adults [9] relative to that recommended in the general population (0.83 g/kg body weight/day) [90]. However, it is worth noting that the amount of protein needed in the relapsing-remitting course, notably in the period of MH remains to be determined and might be above the recommended daily allowance for proteins. In fact, because of the different symptomatology between CD and UC, protein needs during the remission phase may also differ, but new studies are required to document this aspect. The differences between the few studies that have evaluated the efficiency of dietary protein supplementation (composition of the diet, delivery route, duration, MH criteria) during UC remission periods [89,91] lead to different approaches between clinicians. The body of recommendations is generally to follow a diversified and a well-balanced diet. However, it is pretty common to see IBD patients avoiding some foods as a way to decrease relapse risk [92]. In fact, after a mucosal injury, the MH process is triggered as a chain of proteino-energetically expensive mechanisms that aim to restore the continuity of the tissue [93,94]. The need for dietary proteins may then depend on the level of protein synthesis in the wounded area. 

In addition to their role as building blocks for protein synthesis, AA play essential functions in intestinal homeostasis since they can be used as energy substrates, for cell signaling, and for the production of bioactive nitrogenous compounds such as nitric oxide and glutathione [95]. Metabolomic studies revealed an alteration of AA metabolism in the serum and urine of IBD patients [52]. Indeed, the analysis of the plasma aminogram has been shown to be an effective tool to diagnose and monitor disease activity, notably according to histidine and tryptophan concentrations that are negatively correlated with C-reactive protein in UC and CD patients [96]. These observations suggest that restoring AA supply and metabolism might be a promising adjuvant of a therapeutic approach in IBD. 

In this context, a substantial number of studies aimed to evaluate the effects of AA supplementation in animal models of colitis or in IBD patients, while very few studies investigated the efficiency of dietary protein intake in such models. AA supplied in a free form (as opposed to part of a protein) are fully absorbed in the small intestine and would therefore act either locally in the epithelium through the luminal side or via the bloodstream to reach mucosal sites distant from the AA absorptive sites. Dietary interventions with specific AA in IBD models revealed convincing effects of AA supplementation on the modulation of the inflammatory responses, oxidative stress reduction, and anti-apoptotic effects in the gut, as recently reviewed by Zhang et al. [97]. Several AA in combination or alone showed encouraging effects in attenuating intestinal inflammation and restoring intestinal mucosa homeostasis. 

### 4.1. Effect of Amino Acid Supplementation on Intestinal Inflammation Resolution

In the rat model of colitis treated with Dextran Sodium Sulfate (DSS), supplementations with threonine, serine, proline, and cysteine or with threonine and cysteine only, promote mucin synthesis and increased mucin secretion, thus favoring colonic protection and repair [43,98]. Another study performed in the same model has shown that the combination of threonine, methionine and glutamate supplementation ameliorates colonic MH but not inflammatory status [99]. Although the mechanisms of action by which the three AA accelerate the colonic MH remain unclear, it can be presumed, as already discussed above, that these AA may serve as building blocks for macromolecule synthesis in the wounded mucosal area, as energy substrates for anabolic pathways, and as precursors for bioactive metabolites [100].

Glutamine supplementation is probably the dietary approach that has been the most studied in IBD models (reviewed in [101]), probably firstly because this AA is one of the main energy substrate for IEC [102] and secondly because its mucosal concentration is decreased in IBD [103]. Most of the animal inflammatory studies showed beneficial effects of this AA, that might be related to a reinforcement of tight-junctions, an increase protein synthesis, a support for energy metabolism and a modulation of oxidative stress and inflammation [101]. In addition, oral pre-treatment with glutamine in rats that underwent acetic acid intestinal injury, resulted in a lower score of intestinal mucosa damage and a concomitant stimulation of cell turnover (enhanced proliferation and reduced apoptosis) [104]. Similar results were obtained in a rat model of colitis induced by trinitrobenzene sulphonic acid (TNBS), with rectal instillation of glutamine that prevents colonic fibrosis development, a common complication in CD. Such results in rats with colitis and treated with glutamine were associated with the downregulation of the increased expression of several gene pathways that contribute to the accumulation of matrix proteins [105]. However, there is limited evidence of the beneficial effects of glutamine supplementation in humans. For example, a trial performed with active CD patients failed to show any advantage of a glutamine-enriched polymeric diet compared to a standard diet [106].

In a porcine model of colitis, tryptophan supplementation improved clinical symptoms and barrier function, and reduced pro-inflammatory cytokine expressions [107]. Similar results were obtained in a mouse model of colitis in which tryptophan supplementation improved clinical parameters and decreased nitrosation in the colon [108]. One potential mechanism for such an effect is the regulation by dietary tryptophan of antimicrobial peptide expression via the mTOR pathway in IEC that consequently regulates the gut microbiota composition [109]. Moreover, tryptophan is converted to kynurenine by the 2,3-dioxygenase (IDO) that is overexpressed in the epithelium during colitis and contributes to limit the inflammatory process [110]. In TNBS-induced colitis rats and in DSS-treated mice, l-arginine supplementation reduced colonic inflammation [111,112,113]. Additionally, l-arginine treatment of DSS-treated mice resulted in increased ex vivo migration of colonic epithelial cells, suggestive of an increased capacity for wound repair [114]. In a pig model of colitis, cysteine decreased inflammation and intestinal permeability [115]. Knowing that cysteine is the rate-limiting substrate for the synthesis of glutathione, it may play a role in the control of oxidative stress through the production of this tripeptide. Histidine has beneficial effects in an experimental murine model of colitis through histamine production and the inhibition of inflammation [116]. Glycine supplementation also showed a beneficial effect by inducing immunoregulation [117]. Moreover, *poly*-l-lysine showed anti-inflammatory effects in the intestinal mucosa that are primarily mediated by the activation of the calcium-sensing receptor [118].

In conclusion, some AA have been shown to exert beneficial effects on colitis. However, in most studies, AA supplementation was performed before colitis induction (preventive approach) or during colitis induction. The optimal timing for AA supplementation in the course of IBD, particularly during inflammatory resolution, remains to be determined at least in experimental models and ideally in humans. Regarding this latter point, it is worth noting that the effects of nutritional intervention in IBD patients may prove to be difficult to evaluate, taking into account the heterogeneity of patients with different clinical history and treatment.

### 4.2. Effect of High-Protein Diets on Colitis Resolution

In animal studies, colitis has been shown to be associated with a marked increase in the protein fractional synthesis rate in the colon [99,119,120,121]. In addition, in a mice model of colitis, an HP intake (whole milk protein), although increasing the mortality of animals when compared to an isoenergetic NP diet, displayed beneficial effects on the surviving animals during the colitis remission phase [45]. Indeed, the HP diet induced a thickening of the colonic epithelium during the resolution phase concomitant with an increase in the promoting epithelial repair cytokine TGF-β3, an increased epithelial proliferation, and a remodeling of the expression of tight-junction proteins [45]. Inversely, a protein-free diet worsened DSS-induced colitis in mice [109]. 

These data suggest that the quantity and the source of protein might influence colitis evolution in animal models, but more studies are obviously needed to identify the optimal protein intake during IBD. For that purpose, it is essential to unravel the mechanisms through which protein intake might influence positively or negatively the IBD pathogenesis.

## 5. Conclusions and Perspectives

The use of dietary proteins and AA supplementation to control inflammation and promote MH, although being suggested in experimental models, awaits further human studies in order to establish its possible interest in clinical practice. Protein intake, according to the protein source, might indeed influence intestinal health through two mechanisms; (i) modulation of the quantity and nature of AA absorbed and supplied to intestinal tissues through the bloodstream and (ii) modulation of the quantity and nature of undigested proteins delivered to the large intestine. Although epidemiological evidence indicates that the consumption of a diet with high animal protein content is associated with an increased risk of IBD, the mechanisms which would explain such an association remain elusive. Table 2 recapitulates some typical effects of a diet with a high animal protein content on the colonic ecosystem in studies with experimental models. One interesting hypothesis is based on the effects of some AA-derived bacterial metabolites, which have been shown to exert deleterious effects on the colonic epithelial cells when present in excess towards their metabolic capacity for detoxication. In contrast, supplementation of the diet with selected AA may have beneficial effects. New information regarding the needs for dietary nitrogenous compounds with different AA contents in both the active phase of IBD and the phase of MH are clearly needed. In addition, little is known of the role of proteins originating from plants for IBD therapy and associated potential benefits, since most of the studies have been performed with animal proteins. Finally, the complete MH and its induction via nutritional therapy with a correct amount of chosen dietary proteins or supplemental AA remains a field of possibilities. Then, future experimental, clinical, and epidemiological works are needed to evaluate the quantity and quality required for the best efficiency. The ultimate goal is the development of successful patient-tailored diets for IBD patients to optimize clinical therapeutic responses while reducing adverse effects.

## Figures and Tables

**Table 1:** Some major metabolites produced from amino acids by the microbiota.

Precursor	Bacterial Metabolite
All amino acids	Ammonium
Valine	Isobutyrate
Isoleucine	2-methylbutyrate
Leucine	Isovalerate
Tryptophan	Indole
Tyrosine	*p*-cresol, phenol
Phenylalanine	Phenylacetate
Lysine	Cadaverine
Cysteine	Hydrogen sulfide

**Table 2:** Typical effects of a high animal protein diet on the colon in experimental model studies.

Microbiota composition	↘ *F. prausnitzii* abundance and *Clostridium coccoides* and *C. leptum* groups [22,23]
	↘ bacterial diversity [22]
	↗ *Escherichia/Shigella*, *Enterococcus*, *Streptococcus*, and sulfate-reducing bacteria abundance [23]
Microbiota metabolic activity	↗ protease activity [31]
Luminal environment changes	↗ water content [31]
	↗ amino acid-derived bacterial metabolite amounts [31,65]
Colonic epithelium	↘ energy metabolism efficiency [31]
	↘ tight junction protein expression [35]
	↘ colonocyte brush-border height [31]
	↘ butyrate transporter expression [50]
	↗ detoxification enzymes of amino acid-derived bacterial metabolites [31,65]
	↗ expression of genes involved in cell proliferation and barrier function [36]
	↘ expression of genes involved in cell metabolism, NF-κB signaling, DNA repair, glutathione metabolism, and cellular adhesion [36]
	↗ expression of mucin gene expression, and alters goblet cell distribution in the epithelium [36,40,43]

The symbols ↘ and ↗ mean decrease in and increase in, respectively.

## Abbreviations

AA	amino acid
CD	Crohn’s disease
DSS	Dextran Sodium Sulfate
HP	high-protein
IBD	Inflammatory bowel diseases
IEC	intestinal epithelial cells
TNBS	trinitrobenzene sulphonic acid
UC	ulcerative colitis
